# Correlation of perinatal outcomes with amniotic fluid assessment techniques in high-risk pregnancies in a Tertiary Hospital in Southern Nigeria

**DOI:** 10.4314/ahs.v21i3.42

**Published:** 2021-09

**Authors:** Ovoke Egagifo, Lawrence O Omo-Aghoja, Ayotunde T Adeyinka

**Affiliations:** 1 Calderdale and Huddersfield NHS Foundation Trust, Obstetrics and Gynecology Huddersfield, West Yorkshire, UK; 2 Department of Obstetrics and Gynecology, Faculty of Clinical Medicine, College of Health Sciences, Delta State University, Abraka, Nigeria; 3 Department of Obstetrics and Gynecology, Delta State University Teaching Hospital, Oghara, Nigeria

**Keywords:** Two-diameter pocket, amniotic fluid index, oligohydramnios, high-risk pregnancies, adverse perinatal outcomes

## Abstract

**Background:**

Oligohydramnios is a predictor of fetal compromise and a useful tool in pregnancy management. It has been assessed using various techniques, including two-diameter pocket (2-DP) and amniotic fluid index (AFI).

**Objectives:**

To determine which of these two techniques best diagnose oligohydramnios and predicts adverse perinatal outcomes.

**Methods:**

This was a comparative cross-sectional study conducted at Delta State University Teaching Hospital, Oghara in southern region of Nigeria over eight months period. One hundred high-risk pregnant women were recruited and ultrasound determination of amniotic fluid was performed using AFI and 2-DP. The women were followed up till delivery to determine adverse perinatal outcomes.

**Results:**

The indices of validity of AFI and the 2-DP were calculated and compared. The 2-DP had a higher sensitivity than AFI for adverse outcomes in high-risk pregnancies complicated by oligohydramnios.

**Conclusion:**

The 2-DP technique should preferably be used for the assessment of oligohydramnios in high-risk pregnancies.

## Introduction

Amniotic fluid (AF) not only provides physical protection, space for movement and growth, and enhancement of lung development for the fetus but it also constitutes a vital sign for the evaluation of fetal well-being.^1^,^2^ The metabolism of AF is a fine dynamic balance between the fetus and the mother.^1^ Among its physical characteristics, its volume is the most important index utilized in ante-partum fetal surveillance to determine fetuses at high risk of compromise, and requiring interventions to improve perinatal outcome.

Consensus on a gold standard of amniotic fluid volume (AFV) measurement is lacking.^3^–^5^ Direct measurement at Cesarean delivery and dye dilution technique are more accurate but more cumbersome than ultrasonographic measurement.^6^–^8^ Ultrasound measurement therefore remains the only practical method and the techniques employed are amniotic fluid index (AFI), two-diameter pocket (2-DP), and single deepest pocket (SDP).^7^

The amniotic fluid index (AFI) is determined by the summation of the vertical diameter of the largest pocket in each of the uterine cavity's four quadrants^9^ and values of 5–25 cm^2^ are considered normal.^10^ The 2-DP on the other hand is the product of the depth and width of the largest single pocket free of fetal or umbilical parts; and normal value is 15–50 cm.^1^,^2^ From the foregoing description, it is obvious that it is easier to undertake the 2-DP assessment both in terms of learning the requisite skills and in conduct of the needed scanning procedure. It is also instructive to note that this is a level II ultrasound scan procedure and can be done with 2-D obstetric ultrasound. And for this study, a Toshiba machine with a 3.5 MHz transducer was used.

The 2-DP technique has been suggested to be more accurate than AFI at detecting oligohydramnios^11^ because it has higher sensitivity and negative predictive values.^6^,^12^,^13^ It is however associated with a high false-positive rate.^11^ Studies on the correlation between 2-DP and adverse perinatal outcomes are sparse. The AFI has been more widely studied, and the findings suggest poor correlation with adverse perinatal outcomes.^6^,^14^,^15^ These studies were conducted using multiple sonographers which could have introduced bias. The conclusion on which is better between the 2-DP and AFI for the prediction of perinatal outcomes is yet to be made.^3^ Finding the more effective AFV determinant that better predicts adverse perinatal event will improve perinatal outcomes in high-risk pregnancies. The aim of this study was to determine the better predictor of adverse perinatal outcomes among high-risk pregnant women with oligohydramnios.

## Materials and Methods

This was a comparative cross-sectional study that compared the indices of validity for AFI and the 2-DP among high risk black African pregnant women. It was conducted among women attending the antenatal clinic of the Obstetric unit of Delta State University Teaching Hospital (DELSUTH), Oghara, over a duration of 8 months from 5th June, 2018 to, 4^th^ February, 2019. The sample size (n = 100) was calculated using the formula n = z2 p q / d2,^16^,^17^ where z = area under normal curve corresponding to 95% confidence interval of 1.96; p = prevalence of oligohydramnios among high risk pregnancies = 0.06 (6%);^18^ and d = desired precision of 0.05 (acceptable error in the estimation for 95% confident interval) with adjustment factor for attrition rate of 10%.

The study population comprised high risk pregnant women at 37 weeks to 41 weeks +6 days with known gestation age (by date or early USS) with intact membranes. The high-risk factors considered were hypertensive disorders in pregnancy, anemia in pregnancy, and gestational diabetes mellitus. Only those who delivered within 7 days of AFV assessment were included in the analysis. Women with multiple gestation, breech presentation, anteriorly sited placenta, and fetal anomalies were excluded. Eligible participants were recruited consecutively (this was expedient as it helped achieve the timeline for this study) from the antenatal clinics and labour ward, after they had been counselled, and written informed consent obtained.

All participants had 2-D obstetric ultrasound scan done in the fetal assessment unit using a Toshiba machine and 3.5 MHz transducer. Measurement were taken in centimetres in the vertical plane, with patients lying supine, and transducer held parallel to their longitudinal axis and perpendicular to the floor. The AFI was determined according to method described by Phelan^9^ where the maternal abdomen is divided into four quadrants using the umbilicus and the linea nigra as reference markers, and the deepest pool in each quadrant measured. AFI was derived by the sum of the four measurements. The largest vertical and transverse diameters of the largest fluid pocket were also measured, and 2-DP was derived by multiplying the two diameters. Measurements were taken from pools free of umbilical cord or fetal parts, and the average of the 3 readings taken. Oligohydramnios was defined as AFI ≤ 5.0 cm, normal AFI as 5–25cm, and polyhydramnios as AFI > 25cm. For the 2-DP, oligohydramnios was defined as < 15 cm^2^, normal value was 15– 50cm^2^ and polyhydramnios was > 50cm.^2^ ^11^,^19^ All participants were monitored till delivery, and perinatal outcomes (fetal death, non-reassuring fetal heart pattern, econium staining of the liquor, mode of delivery other than SVD, low 5^th^-minute Apgar score (i.e less than 7), meconium aspiration, need for neonatal resuscitation, NICU admission, prolonged length of NICU admission, early neonatal death). Findings for each participant were recorded on a data collection sheet.

The completed data collection sheets (proforma) were collated, coded and entered into the computer using Statistical Package for Social Scientist (SPSS version 21, Armonk, NY SPSS Inc., Chicago Ill, USA). The data was analyzed using the same Statistical Package for Social Scientist and this consisted of univariate analysis and comparisons of identified relationships. Tests of statistical significance were based on 95% confidence interval using Chi square test with Yates or Fischer Exact correction where applicable. The performance of the AFI and the 2-DP in predicting adverse perinatal outcomes were determined by evaluating and comparing their indices of validity using chi-square tests, at a 95% confidence interval. P values <0.05 were deemed significant. Odds ratio and confidence was then calculated to identify independent factors associated with adverse perinatal outcomes among high-risk pregnant women with oligohydramnios.

The study was approved by the Health Research and Ethics Committee of the Delta State University Teaching Hospital, Oghara.

## Results

The mean age of the hundred women that participated in the study was 30.76 ± 7.77 years. Most (44%) of them were primigravidae. The commonest high-risk conditions were hypertensive diseases and this was the situation in 73.0% of the cases recruited. Four-fifths (81%) of the women had early term deliveries (table 1).

**Table 1:** Socio-clinico-demographic characteristics of Study Participants

Variables	Categories	Frequency N= 100 (%)
**Age in years**	15–19	7 (7.0)
	20–24	16 (16.0)
	25–29	20 (20.0)
	30–34	24 (24.0)
	35–39	17 (17.0)
	40–44	11 (11.0)
	≥ 45	5 (5.0)
	**Mean ± SD**	30.76 ± 7.77
**Parity**	Primigravida	44 (44.0)
	P1–P4	42 (42.0)
	Grand multipara	14 (14.0)
	**Mean=1.93 ± 2.19**	
**High Risk conditions**	PIH	32 (32.0)
	Preeclampsia	29 (29.0)
	Chronic HTN	12 (12.0)
	GDM	5 (5.0)
	Anemia	18 (18.0)
	Infection	4 (4.0)
**Gestational age at Delivery**	37–38^+6^	81 (81)
	39–40^+6^	17 (17)
	41–41^+3^	2 (2)

More than three-quarters (77.0%) of the deliveries required interventions, and of these, 38.0% were cesarean sections on account of fetal distress. In about 29.0% of cases the Apgar score was poor at the 5th minute of life, and more than half (58.0%) of the babies required neonatal intensive care units (NICU) admission for various indications (Table 2).

**Table 2:** Perinatal Outcomes

OUTCOMES	Categories	Frequency (%) N =100
**Adverse Intrauterine** **outcomes**	Fetal distress	46 (46.0)
	IUFD	11 (11.0)

**Modes of delivery**	AVD with fetal distress	8 (8.0)
	AVD without fetal distress	3 (3.0)
	Elective	15 (15.0)
	Emergency with fetal distress	38 (38.0)
	Emergency without fetal distress	13 (13.0)
	SVD	23 (23.0)

**Birth-weight distribution**	<2,500g (LBW)	35 (35.0)
	2,500–3,999g	49 (49.0)
	≥4000g (Macrosomia)	16 (16.0)

**Meconium stained liquor**	Fresh Meconium	15 (15.0)
	Stale Meconium	19 (19.0)

**Low APGAR Score**	Poor at 1^st^minute	65 (65.0)

	Poor at 5^th^ minute	29 (29.0)

**Adverse Post-natal** **outcomes**	Prolonged LOS	32 (32.0)
	NICU admission	58 (58.0)
	ENND	6 (6.0)
	RDS	17 (17.0)

There was 3-fold more likelihood to report normal amniotic volume with AFI technique as compared to 2-DP (p<0.05; OR=3.30, CI: 1.85–5.89). On the other hand, the 2-DP technique was more likely to report oligohydramnios than AFI (P <0.01); (OR=0.26, CI: 0.15–0.47) (Table 3).

**Table 3:** Amniotic fluid values by AFI and 2-DP techniques

Techniques	AFV		Total	Odds Ratio (OR)	*X* ^2^	P-value	95% CI	
	
	Normal	Abnormal					Lower	Upper
AFI	64(64.0)	36(36.0)	100(100.0)					
2-DP	35(35.0)	65(65.0)	100(100.0)	3.30	16.82	<0.01	1.85	5.89

	**Oligohydramnios**						
							
	Present	Absent						

AFI	29(29.0)	71(71.0)	100(100.0)					
2-DP	61(61.0)	39(39.0)	100(100.0)	0.26	20.69	<0.01	0.15	0.47

Abnormal subsets of AFI categories were thrice more likely to predict an adverse outcome compared to a normal subset of AFI category (OR=3.08; CI=0.82–11.55). The 2-DP technique correctly predicted more adverse perinatal outcomes than normal outcomes (89.2% versus 28.6%), this association was statistically significant (p<0.05). Indeed, abnormal subsets of 2-DP categories were thrice more likely to predict an adverse outcome compared to a normal 2-DP category (OR=3.31; CI=1.13–9.70) (Table 4). Of the 33 abnormal cases by AFI, 29 had oligohydramnios and 3 had polyhydramnios, however cases of polyhydramnios were not considered further as it was not the focus of this study. Of the 61 babies with abnormal fluid category (table 3), it was 58 of these babies with abnormal fluid categories that had adverse outcomes.

**Table 4:** Amniotic fluid categories by AFI and 2-DP and perinatal outcomes

Techniques		Perinatal Outcomes		
		Frequency (%)		
**AFI category**		**Adverse**	**Normal**	**Total**
	**Abnormal**	33 (91.7)	3 (8.3)	36 (100.0)
	**Normal**	50 (78.1)	14 (21.9)	64 (100.0)
	**Total**	83 (83.0)	17 (17.0)	100 (100.0)

**2-DP category**	**Abnormal**	58 (89.2)	7(10.8)	65 (100.0)
	**Normal**	25 (71.4)	10 (28.6)	35(100.0)
	**Total**	83 (83.0)	17 (17.0)	100 (100.0)

*AFI X^2^*= 3.00, P = 0.08, SN = 39.76%; SP =82.35%; Accuracy = 47%; PPV =91.67%; NPV= 21.87%; OR = 3.08(CI:0.82–11.55); LR(+) = 2.25; LR(-)=0.73

*The 2-DP* X^2^ = 3.93, P = 0.05; SN=69.88%; SP= 58.82%; PPV= 89.2%; NPV= 28.6%; AE= 18; RE=21.69%; OR=3.31 (CI: 1.13–9.70); Accuracy = 68%; LR (+) =1.70; LR (-) = 0.51

Table 5 below compares the labour outcomes of patients with oligohydramnios by AFI and 2-DP respectively. Then selected outcomes displayed in the table 6 were culled from this table to compute components therein.

**Table 5:** Labour outcomes for patients who had oligohydramnios by AFI versus 2-DP

OUTCOMES	Categories	Technique		*X* ^2^	P value	OR	95% CI	
							
		AFI (29)	2-DP (61)				LL	UL
**Intrauterine** **Events**	Fetal distress	18 (62.07)	28(45.90)	2.06	0.15	1.93	0.78	4.76
	IUFD	5 (17.24)	9(14.75)	0.09	0.76+	1.20	0.36	3.98

**Mode of delivery**	AVD Fetal Distress	3 (10.34)	8(13.11)	0.14	1.00	0.76	0.19	3.12
	EMCS Fetal Distress	19 (65.52)	38(62.30)	0.09	0.77	1.15	0.46	2.90
	SVD	7 (24.14)	15(24.59)	0.05*	0.83	0.98	0.35	2.74

**Birth-weight** **distribution**	<2,500g	16 (55.17)	27(44.26)	0.94	0.33	1.55	0.64	3.77
	2,500–3,999g	11 (37.93)	29(47.54)	0.74	0.39	0.67	0.27	1.66
	≥4000g	2 (6.9)	5(8.2)	0.04*	0.84	0.83	0.15	4.56

**Meconium** **stained liquor**	Fresh Meconium	6 (20.69)	8(13.11)	0.38*	0.54	1.73	0.54	5.55
	Stale Meconium	11(37.93)	16(26.23)	1.28	0.26	1.72	0.67	4.41

**Low APGAR** **Score**	Poor at 1^st^ minute	23(79.31)	45(73.77)	0.10*	0.76	1.36	0.47	3.95
	Poor at 5^th^minute	11(37.93)	20(32.79)	0.23	0.63	1.25	0.50	3.15

**Adverse Postnatal** outcomes*	Prolonged LOS	12(41.38)	19(31.15)	0.91	0.34	1.56	0.62	3.9
	NICU admission	23(79.31)	38(62.30)	2.61	0.15+	2.32	0.82	6.54
	ENND	3(10.34)	4(6.56)	0.12*	0.73	1.86	0.39	8.96
	RDS	4(13.79)	6(9.84)	0.04*	0.84	1.47	0.38	5.66

*Multiple responses +Fisher's exact *Yates correction; CI: confidence interval

The 2-DP had higher sensitivities than that of AFI in the detection of adverse perinatal outcomes (69.88% versus 39.76%), p < 0.01; (OR=0.29; CI: 0.16–0.51); predict fetal distress (75% versus 50%) (P <0.01) (OR=0.33; 0.18–0.61); but not in predicting the need for resuscitation (24.2% versus 42.4%); p <0.01; (OR=2.29; CI: 1.25–4.21). The specificity of AFI was higher than that of 2-DP technique in predicting the absence of adverse perinatal outcomes (82.35% versus 58.82%); (p <0.01); (OR=3.17; CI: 1.66–6.05); the absence of RDS (66.30% vs. 32.53%); (p <0.01,); (OR = 3.94; CI: 2.19–7.09); prolonged length of stay (LOS) (69.1% vs. 32.29%), p<0.01, (OR=4.13, CI: 1.93–6.17); and absence of ENND (66.0% vs. 36.17%) p<0.01. (OR=3.45, CI: 1.93–6.17) (Table 6).

**Table 6:** Sensitivity and Specificity of AFI and 2-DP for selected outcomes

**Outcomes**	**Sensitivity**		X^2^	**P-value**	**Odds** **Ratio**	**95% CI**	
		
	**AFI≤5cm**	**2-DP<15cm^2^**				**LL**	**UL**

**Adverse Perinatal outcomes**	39.7	69.8	18.18	<0.01	0.29	0.16	0.51
**IUFD**	9.2	62.9	60.96*	<0.01	0.06	0.03	0.13
**Fetal distress**	50.0	75.00	13.33	<0.01	0.33	0.18	0.61
**Need for Resuscitation**	42.4	24.2	7.33	0.01	2.29	1.25	4.21
**NICU admission**	48.3	70.6	10.98	<0.01	0.38	0.21	0.68
**RDS**	47.1	47.0	0.00	1.00	1.00	0.57	1.74
**Prolonged LOS**	46.1	65.6	8.12	<0.01	0.44	0.25	0.78
**ENND**	66.7	83.3	6.83	0.01	0.42	0.21	0.81

	**Specificity**						
	**AFI≤5cm**	**2-DP<15cm^2^**					

**Adverse Perinatal outcomes**	82.3	58.8	12.71	<0.01	3.17	1.66	6.05
**IUFD**	14.2	18.1	0.34	0.56	0.74	0.35	1.59
**Fetal distress**	73.3	41.6	19.66	<0.01	3.73	2.06	6.76
**Need for Resuscitation**	76.5	55.8	8.91	<0.01	2.49	1.35	4.56
**NICU admission**	80.9	57.1	13.46	<0.01	3.21	1.70	6.08
**RDS**	66.3	32.5	21.78	<0.01	3.94	2.19	7.09
**Prolonged LOS**	69.1	35.2	23.16	<0.01	4.13	2.29	7.46
**ENND**	66.0	36.1	18.01	<0.01	3.45	1.93	6.17

LL: lower limit; UL: upper limit

The 2-DP technique had significantly higher PPV than AFI technique in predicting NICU admission (34.2% vs. 63.1%) (p< 0.01), (OR = 0.3, CI: 0.17 – 0.54) and IUFD (20.2% vs 82.2%) (p< 0.01), (OR = 0.04, CI: 0.02 – 0.09). There was no significant difference in the negative predictive value of both techniques for adverse pregnancy outcomes (Table 7).

**Table 7:** Predictive Values of AFI and 2-DP for selected outcomes

**Outcomes**	**AFI≤5cm**	**2DP<15cm^2^**	**X^2^**	**P-value**	**Odds** **Ratio**	**95% CI**	

						**LL**	**UL**

	**PPV**	**PPV**					
**Adverse Perinatal outcomes**	91.67	89.20	0.23*	0.63	**1.42**	0.54	**3.70**
**IUFD**	20.2	86.20	84.81*	<0.01	0.04	0.02	0.091
**Fetal distress**	55.6	41.67	3.92	0.05	1.76	1.00	3.082
**Need for Resuscitation**	77.8	79.9	0.03	0.86	0.89	0.45	1.75
**NICU admission**	34.2	63.1	16.84	<0.01	0.30	0.17	0.54
**RDS**	22.2	13.80	0.11	0.74	0.89	0.46	1.73
**Prolonged LOS**	41.7	32.30	1.66*	0.20	1.73	0.83	3.62
**ENND**	11.10	07.70	0.23*	0.63	1.42	0.55	3.70

	**NPV**	**NPV**					

**Adverse Perinatal outcomes**	21.87	28.60	1.29	0.26	0.69	0.36	1.31
**IUFD**	10.94	5.70	1.03*	0.31	1.94	0.69	5.46
**Fetal distress**	68.8	71.40	0.10	0.76	0.91	0.50	1.67
**Need for Resuscitation**	40.6	54.30	3.39	0.07	0.59	0.34	1.04
**NICU admission**	53.1	51.40	0.08	0.78	1.08	0.62	1.89
**RDS**	85.9	77.10	2.68	0.15+	1.84	0.88	3.82
**Prolonged LOS**	73.4	68.00	0.60	0.44	1.27	0.69	2.34
**ENND**	96.9	97.10	0.17	0.68*	1.00	0.20	5.08

+Fisher's exact *Yates correction

The likelihoo d ratio (+) as predicted by AFI technique was higher than that of 2-DP technique for the occurrence of fetal distress (1.87 vs. 1.29) p<0.01 (OR=2.35, CI:1.34–4.10); need for resuscitation (1.79 vs. 0.55), p<0.01, (OR=3.20, CI:1.58–6.50); and occurrence of RDS (1.40 vs. 0.70), p<0.05, (OR=2.00 CI:1.12–3.56). There was no statistically significant difference between the likelihood ratios (-) of AFI and 2DP of correctly predicting the absence of adverse perinatal outcomes, (0.73 vs. 0.51) p=0.19(OR=1.44 CI:0.83–2.50). The 2-DP had a significantly higher negative likelihood ratio compared to the AFI for IUFD, need for resuscitation, and RDS. AFI has higher LR (-) for fetal distress (Table 8).

**Table 8:** Positive and Negative Likelihood Ratio of AFI and 2-DP for selected outcomes

Outcomes	AFI≤5cm	2DP<15cm^2^	X^2^	P value	OR	95% CI*	

						LL	UL

POSITIVE LR	LR (+)	LR (+)					
**Adverse Perinatal outcomes**	2.25	1.70	0.55	0.45	1.30	0.66	2.56
**IUFD**	6.35	0.77	31.54	<0.01	0.14	0.06	0.29
**Fetal distress**	1.87	1.29	9.18	<0.01	2.35	1.34	4.10
**Need for Resuscitation**	1.79	0.55	10.76	<0.01	3.20	1.58	6.50
**NICU admission**	2.53	0.65	1.64	0.20	1.53	0.80	2.94
**RDS**	1.4	0.70	5.60	0.02	2.00	1.12	3.56
**Prolonged LOS**	1.35	1.01	1.81	0.19	1.46	0.83	2.58
**ENND**	1.96	1.31	2.26	0.13	1.50	0.88	2.53

**NEGATIVE LR**	**LR (-)**	**LR (-)**					

**Adverse Perinatal outcomes**	0.73	0.51	0.21	0.19	1.44	0.83	2.50
**IUFD**	0.12	2.04	8.49	<0.01	3.16	1.43	7.01
**Fetal distress**	0.68	0.60	17.58	<0.01	0.14	0.05	0.38
**Need for Resuscitation**	0.75	1.36	5.43	0.02	0.56	0.35	0.91
**NICU admission**	0.64	0.51	0.65	0.42	1.20	0.72	2.22
**RDS**	0.80	1.63	5.84	0.02	0.50	0.28	0.88
**Prolonged LOS**	0.77	0.97	0.67	0.41	0.78	0.43	1.41
**ENND**	0.51	0.46	0.71	0.14	1.14	0.56	2.38

The linear relationship between AFI and 2-DP was positive for all, however it was stronger for overall values (r = 0.75) than for oligohydramnios; the linear relationship was statistically significant (P<0.05) (Table 9).

**Table 9:** Correlation of AFI and 2DP

Category	AFI (mean ± SD)	2DP (mean ± SD)	Pearson's Coefficient (r)	P value
**Overall**	11.76 ± 7.66	14.22 ± 13.90	0.75	<0.01
**Oligohydramnios**	3.16 ± 0.99	2.32 ± 1.62	0.42	0.03

The sensitivity of the 2-DP was higher than that of the AFI. The difference in the sensitivity of AFI and 2-DP was statistically significant (p<0.01). The 2-DP technique was more likely to correctly predict the occurrence of adverse perinatal outcomes than the AFI technique. (OR=0.29, CI: 0.16–0.51). On the other hand, the specificity of the AFI was higher than that of the 2-DP. This was statistically significant, p<0.01. AFI was 3 times more likely to predict the absence of adverse perinatal outcomes compared to the 2-DP technique. (OR=3.17, CI: 1.60–6.05). The 2-DP technique was significantly more accurate in predicting the adverse perinatal outcomes than the AFI technique (68% vs. 47%). P <0.01, OR 0.42 (0.23–0.74). The AFI technique has higher absolute error and relative error than 2-DP technique, with the latter being statistically significant P<0.05, OR= 3.02, CI: 1.63–5.59) (Table 10).

**Table 10:** Validity of assessment techniques

Indices of validity	AFI≤5cm	2-DP<15cm^2^	P-value	Odds ratio	95% CI	

					LL	UL
Overall sensitivity	39.76	69.88	<0.01	0.29	0.16	0.51
Overall specificity	82.35	58.82	<0.01	3.17	1.66	6.05
Overall PPV	91.67	89.20	0.63	**1.42**	0.54	**3.70**
Overall NPV	21.87	28.20	0.26	0.69	0.36	1.31
LR(+)	2.25	1.70	0.45	1.30	0.66	2.56
LR (-)	0.73	0.51	0.19	1.44	0.83	2.50
Absolute error	45	18	27*	-	-	-
Relative error	45.78%	21.69%	<0.01	3.02	1.63	5.59

LL: lower limit; UL: upper limit

* Difference in absolute error

## Discussion

This study was undertaken with a view to determine which of the two techniques – AFI and 2-DP assessment of AFV that better predicts adverse perinatal events with a view to using the findings to design appropriate and relevant interventions that will improve perinatal outcomes in high risk pregnancies. The commonest high-risk condition seen amongst the study subjects was hypertensive disorders of pregnancy which necessitated early delivery in some cases. This is not unexpected as the social and clinical profile analysis shows that primigravidity is the commonest parity group, which is often associated with pregnancy induced hypertension. The ultimate aim of antepartum surveillance tests is the prevention of adverse pregnancy outcomes. At least one adverse perinatal outcome occurred in 83 out of 100 high-risk pregnancies in this study. This is expected in high-risk pregnancies and had been reported by other studies.^20^

This study showed that AFI technique identified more normal volumes (5–25cm) compared to the 2-DP technique (15–50cm2). On the other hand, the 2-DP technique identified oligohydramnios (<15cm^2^) more often than AFI (<5cm). This is consistent with findings by Magann et al^12^,^21^,^22^ where the 2-DP more frequently diagnosed oligohydramnios compared to the AFI. This higher sensitivity translates to higher false positive figures. Obstetric review of parturients against their clinical background still remains the backbone of sound decision making.

Clearly in evidence for this study, is that both amniotic fluid volume assessment techniques were likely to predict adverse perinatal outcomes when there was oligohydramnios compared to when the values were normal. The 2-DP technique did this more than the AFI technique. This is similar to what was reported in 1991 by Ajayi and Soothill.^20^ This is probably because both techniques provide quantitative results that are proportional to actual volumes.^15^ It is well-established that oligohydramnios is associated with a high-risk of adverse perinatal outcomes.^15^

The sensitivity of the 2-DP technique in predicting overall adverse perinatal outcomes and individual outcomes such as IUFD, fetal distress, NICU admission, prolonged length of stay and early neonatal death in all pregnancies with abnormal AFV was significantly higher than that of AFI. The 2-DP has not been subjected to research as often as the AFI.^13^ One study^23^ reported that the 2-DP <15cm^2^ more frequently correctly predicted Apgar scores<7 at 1 minute than AFI. However, this has not been corroborated by further studies. The AFI performed poorly as predictor of ill fetuses. This study revealed that despite the statistically significant association with adverse outcomes, the AFI had a poor sensitivity for adverse perinatal outcomes. This has been demonstrated by findings from other studies.^11^,^24^,^25^,^26^ Morris et al^26^ observed a statistically significant association of the AFI with adverse outcomes but noted a low sensitivity for major adverse outcomes, fetal distress in labour and admission to the neonatal unit. This also suggests that the routine use of the AFI technique may lead to increased interventional deliveries and thus increase the Cesarean section rate in hospitals. The AFI sensitivity in this study is at variance with findings by other authors.^27^,^28^ On the other hand, Chauhan et al^18^,^29^ had in 1997 reported no significant difference between the ability of both techniques to predict fetal distress. This disparity may be attributed to inter-observer errors on ultrasound scan, efficient management for fetal distress, and the heterogeneity of study participants and populations.

The AFI technique had a higher specificity for adverse perinatal outcomes compared to the 2-DP technique. This was also evident in individual outcomes (fetal distress, need for resuscitation, NICU admission, RDS, prolonged LOS, and ENND). The higher specificity (true negative rate) of the AFI means that this technique is better at identifying normal AFV associated with normal perinatal outcomes. At best, it reassures the Obstetrician of the unlikelihood of an adverse perinatal outcome in the management of a high-risk pregnancy. This is collaborated by other reports.^24^ Threfore, a normal AFI is more useful to the Obstetrician than an abnormal AFI reading that suggests oligohydramnios because of the poor sensitivity.

There was no statistically significant difference between the PPV of 2-DP and AFI for overall adverse perinatal outcomes. However, the 2DP was more likely than AFI to accurately predict IUFD, and NICU admission. The NPV of both techniques for adverse outcomes were low and showed no statistically significant difference. Although this may imply that the ability of each technique to truly rule out an adverse outcome was poor, this statistical test has an inherent weakness of being affected by the prevalence of an outcome. Indeed, the predictive value of any test depends on the prevalence of that condition in the study population.

The AFI technique had a higher positive likelihood ratio compared to 2-DP of accurately predicting the occurrence of IUFD, fetal distress, need for resuscitation, and RDS. This findings mirrors those of Fischer's^30^ where AFI was compared to largest vertical pocket. The 2-DP had a significantly higher negative likelihood ratio compared to the AFI for individual adverse perinatal outcomes (IUFD, need for resuscitation, and RDS. The low sensitivity of the AFI and negative likelihood ratio reveal that a negative test is poor at discriminating correctly between those who will and those who will not have the adverse perinatal outcome.^26^

There was a statistically significant difference between the accuracies and relative accuracy errors for AFI and 2-DP. The 2-DP performed better compared to the AFI in identifying parturients who would develop adverse perinatal outcomes. It was also less prone to errors compared to the AFI. A statistically significant correlation was identified between AFI and 2-DP measurements for overall values and for oligohydramnios, as described in other studies. This is not unexpected as both techniques are designed to measure AFV. Ajayi and Soothill (1991)^20^ reported a significant correlation between AFI and 2-DP.

## Conclusion

This study identified an association between amniotic fluid assessment techniques and adverse perinatal outcomes. It highlights the higher sensitivity and accuracy of the 2-DP technique over the AFI. This study also shows that the 2-DP technique is less prone to errors in predicting adverse perinatal outcomes. The 2-DP appears to be a better tool in predicting adverse pregnancy outcomes. This has important implication on patient care and healthcare policy: it can be suggested that a strong consideration be given to the utilization of-DP technique in the evaluation of amniotic fluid volume in high-risk pregnancies since it better predicts adverse perinatal outcomes in the babies.

One of the strengths of this study is the fact that the level of inter-observer's error is minimal as the sonographic procedures for all the participants were conducted by one operator. The study was not without its limitations. Consecutive rather than randomised recruitment of participants may have introduced bias. The level and quality of evidence for the findings in this study remain limited by the cross-sectional design of the study. This study was conducted among high-risk obstetric population, with disproportionately higher numbers of primigravidae and women with hypertensive disorders in pregnancy. Therefore, there is limitation to how well the findings can be extrapolated to the entire obstetric population.

Future study design should be a randomized controlled trial, which will enable a more rigorous comparison of two-diameter pocket (2-DP) and amniotic fluid index (AFI) techniques, and provide a higher level of evidence as to which of the two is superior in predicting adverse outcome. The outcome will also have better external validity, allowing for more generalizability.
